# Supplementary material for: MicroRNA-mRNA networks define translatable molecular outcome phenotypes in osteosarcoma
Source: Sci Rep. 2020 Mar 10;10:4409. doi: 10.1038/s41598-020-61236-3 (PMC7064533; doi:10.1038/s41598-020-61236-3)
Supplement: Supplementary file 1 — Supplementary Information . [file 41598_2020_61236_MOESM1_ESM.pdf]

**MicroRNA-mRNA networks define translatable molecular outcome phenotypes in osteosarcoma.**

Christopher E. Lietz[1], Cassandra Garbutt[1†], William T. Barry[2], Vikram Deshpande[3], Yen-Lin Chen[4], Santiago A. Lozano-Calderon[1], Yaoyu Wang[2,5‡], Brian Lawney[2,5‡], David Ebb[6], Gregory M. Cote[7], Zhenfeng Duan[8], Francis J. Hornicek[8], Edwin Choy[7], G. Petur Nielsen[3], Benjamin Haibe-Kains[9], John Quackenbush[2,5‡], Dimitrios Spentzos[1]*.

[1]Department of Orthopaedic Surgery, Massachusetts General Hospital, Harvard Medical School, Boston, MA.

[2]Department of Biostatistics and Computational Biology, Dana Farber Cancer Institute, Harvard Medical School, Boston, MA.

[3]Department of Pathology, Massachusetts General Hospital, Harvard Medical School, Boston, MA.

[4]Department of Radiation Oncology, Massachusetts General Hospital, Harvard Medical School, Boston, MA.

[5]Department of Biostatistics, Harvard School of Public Health, Boston, MA.

[6]Pediatric Hematology-Oncology, Massachusetts General Hospital, Harvard Medical School, Boston, MA.

[7]Department of Hematology/Oncology, Massachusetts General Hospital, Harvard Medical School, Boston, MA.

[8]Department of Orthopaedic Surgery, UCLA, Los Angeles, CA.

[9]Department of Medical Biophysics, Princess Margaret Cancer Centre, University of Toronto.

*To whom correspondence should be addressed: Dimitrios Spentzos (dspentzos@mgh.harvard.edu).

† Currently at Illumina, Inc.

‡ Previously at the Dana Farber Cancer Institute.

**Supplementary materials**

**Table S1.** 5-miRNA and 22- miRNA profiles (and the secondary 27 miRNA profile) mapped to each dataset.

| **profile** | **Pilot** [7] | **TARGET** | **MGH** |
|---|---|---|---|
| 5-miRNA | hsa-miR-495-3p | hsa-miR-495-3p | hsa-miR-495-3p |
| | hsa-miR-487b-3p | hsa-miR-487b-3p | hsa-miR-487b-3p |
| | hsa-miR-329-3p | hsa-miR-329-3p | hsa-miR-329-3p |
| | hsa-miR-410-3p | hsa-miR-410-3p | hsa-miR-410-3p |
| | hsa-miR-664a-3p | hsa-miR-664a-3p | hsa-miR-664a-3p |
| 22miRNA | HS_276.1 | - | - |
| | hsa-miR-10b-5p | hsa-miR-10b-5p | hsa-miR-10b-5p |
| | hsa-miR-125b-5p | hsa-miR-125b-5p | hsa-miR-125b-5p |
| | hsa-miR-139-5p | hsa-miR-139-5p | hsa-miR-139-5p |
| | **hsa-miR-323a-3p** | **hsa-miR-323a-3p** | **hsa-miR-323a-3p** |
| | **hsa-miR-329-3p** | **hsa-miR-329-3p** | **hsa-miR-329-3p** |
| | hsa-miR-335-3p | hsa-miR-335-3p | hsa-miR-335-3p |
| | **hsa-miR-337-3p** | **hsa-miR-337-3p** | **hsa-miR-337-3p** |
| | **hsa-miR-376a-3p** | **hsa-miR-376a-3p** | **hsa-miR-376a-3p** |
| | **hsa-miR-379-3p** | **-** | **hsa-miR-379-3p** |
| | **hsa-miR-410-3p** | **hsa-miR-410-3p** | **hsa-miR-410-3p** |
| | **hsa-miR-411-3p** | **hsa-miR-411-3p** | **hsa-miR-411-3p** |
| | **hsa-miR-432-5p** | **hsa-miR-432-5p** | **hsa-miR-432-5p** |
| | **hsa-miR-485-3p** | **hsa-miR-485-3p** | **hsa-miR-485-3p** |
| | **hsa-miR-487b-3p** | **hsa-miR-487b-3p** | **hsa-miR-487b-3p** |
| | **hsa-miR-493-5p** | **-** | **hsa-miR-493-5p** |
| | **hsa-miR-495-3p** | **hsa-miR-495-3p** | **hsa-miR-495-3p** |
| | hsa-miR-514a-3p | - | hsa-miR-514a-3p |
| | hsa-miR-655-3p | hsa-miR-655-3p | hsa-miR-655-3p |
| | **hsa-miR-656-3p** | **hsa-miR-656-3p** | **hsa-miR-656-3p** |
| | hsa-miR-664a-3p | hsa-miR-664a-3p | hsa-miR-664a-3p |
| | **hsa-miR-889-3p** | **hsa-miR-889-3p** | **hsa-miR-889-3p** |

Bolded miRNAs, in addition to hsa-miR-134-5p, hsa-miR-154-3p, hsa-miR-299-5p, hsa-miR-337-5p, hsa-miR-342-3p, hsa-miR-369-3p, hsa-miR-377-3p, hsa-miR-382-5p, hsa-miR-409-3p, hsa-miR-411-5p, hsa-miR-487a-3p, hsa-miR-493-3p, and hsa-miR-539-5p, constitute the 27-miRNA profile previously identified on the 14q32 locus.

**Table S2.** Differential methylation of the CpG probes annotated to the 5-miRNA profile, between the miRNA defined cluster groups. M values are used for the analysis and fold change represents relative methylation of the hypermethylated compared to the hypomethylated group.

| Probe ID | FC | p |
|---|---|---|
| cg14285142 | 1.66 | 0.01187 |
| cg21513316 | 1.55 | 0.0145 |
| cg27074221 | 2.19 | 0.01877 |
| cg02582514 | 1.88 | 0.02497 |
| cg09819642 | 1.58 | 0.02507 |
| cg10177827 | 1.35 | 0.02537 |
| cg00856952 | 1.96 | 0.02817 |
| cg14361526 | 1.58 | 0.03003 |
| cg14910227 | 2.09 | 0.03033 |
| cg21230021 | 1.57 | 0.03573 |
| cg26931307 | 1.85 | 0.03741 |
| cg23270924 | 1.96 | 0.03928 |
| cg21878393 | 1.89 | 0.04104 |
| cg11035122 | 1.68 | 0.0418 |
| cg20547131 | 1.39 | 0.04841 |

**Table S3.** Median Spearman coefficients comparing 5 miRNA profile (the 4 located on 14q32) expression and methylation.

| miRNA | Median Spearman coefficient | Number of probes with $p < 0.05$ |
|---|---|---|
| hsa-miR-329-3p | 0.295 | 32 |
| hsa-miR-410-3p | 0.356 | 36 |
| hsa-miR-487b-3p | 0.361 | 35 |
| hsa-miR-495-3p | 0.345 | 35 |

**Table S4.** Cramer's V and Fisher's exact test results for concordance between the classifications derived from the miRNA signatures and the corresponding gene targets. Cluster and sign average classifications were concordant within each profile (V: 0.294 – 0.715).

| **Groups/classifiers** | | **Fisher's p** | **Cramer's V** |
|---|---|---|---|
| 5miRNA clusters | vs 5miRNA target gene clusters | <0.001 | 0.52 |
| 5miRNA clusters | vs 22miRNA target gene clusters | <0.001 | 0.46 |
| 5miRNA clusters | vs 5miRNA target gene avg model | 0.001 | 0.38 |
| 5miRNA avg model | vs 5miRNA target gene clusters | <0.001 | 0.50 |
| 5miRNA avg model | vs 5miRNA target gene avg model | 0.002 | 0.36 |
| 5miRNA avg model | vs 22miRNA target gene clusters | 0.001 | 0.39 |
| 5miRNA avg model | vs 22miRNA target gene avg model | 0.016 | 0.29 |

**Table S5.** All significantly enriched BioCarta pathways, KEGG pathways, and transcription factor binding sites in the prognostic miRNA gene targets. BioCarta and KEGG pathways with FDR < 0.05 and p < 0.05 for the restrictive 22 and 5-miRNA gene targets, respectively, were considered significant. In addition, 19 enriched KEGG pathways were omitted because they described generic cancer ontology and were considered uninformative (e.g. "Pathways in cancer"). An additional 2 BioCarta and 22 KEGG pathways were enriched in targets of (only) the 5-miRNA profile and 18 BioCarta and 49 KEGG pathways were enriched in targets of (only) the 22-miRNA profile. Transcription factor binding sites were considered significant with FDR < 0.05 for both restrictive gene target lists. The number of gene targets and FDR values presented is for the 22-miRNA profile. An additional 19 transcriptions factor binding sites were enriched in the targets of (only) the 5-miRNA profile, and 147 transcription factor binding sites were enriched in the targets of (only) the 22-miRNA profile. Bolded pathways are perturbed by drugs identified through pharmacogenomic analysis (Table 3).

| BioCarta pathways | Gene targets | FDR |
|---|---|---|
| h_il2rbPathway:IL-2 Receptor Beta Chain in T cell Activation | 12 | 7.46E-05 |
| **h_gleevecPathway:Inhibition of Cellular Proliferation by Gleevec** | 9 | 2.82E-04 |
| h_raccycdPathway:Influence of Ras and Rho proteins on G1 to S Transition | 9 | 1.05E-03 |
| h_igf1rPathway:Multiple antiapoptotic pathways from IGF-1R signaling lead to BAD phosphorylation | 8 | 2.51E-03 |
| h_telPathway:Telomeres, Telomerase, Cellular Aging, and Immortality | 7 | 4.25E-03 |
| h_arfPathway:Tumor Suppressor Arf Inhibits Ribosomal Biogenesis | 6 | 3.40E-02 |
| h_erk5Pathway:Role of Erk5 in Neuronal Survival | 6 | 3.40E-02 |
| h_ptenPathway:PTEN dependent cell cycle arrest and apoptosis | 6 | 3.40E-02 |
| h_tffPathway:Trefoil Factors Initiate Mucosal Healing | 7 | 3.56E-02 |
| h_crebPathway:Transcription factor CREB and its extracellular signals | 7 | 4.35E-02 |
| | | |
| KEGG pathways | | |
| hsa04550:Signaling pathways regulating pluripotency of stem cells | 31 | 3.83E-21 |
| hsa05161:Hepatitis B | 24 | 4.68E-13 |
| hsa04068:FoxO signaling pathway | 23 | 8.47E-13 |
| hsa05166:HTLV-I infection | 29 | 7.70E-12 |
| hsa04012:ErbB signaling pathway | 17 | 4.58E-10 |
| **hsa04151:PI3K-Akt signaling pathway** | 30 | 2.50E-09 |
| hsa04917:Prolactin signaling pathway | 15 | 3.08E-09 |
| hsa04722:Neurotrophin signaling pathway | 18 | 7.95E-09 |
| hsa04015:Rap1 signaling pathway | 21 | 2.76E-07 |
| hsa04915:Estrogen signaling pathway | 15 | 2.88E-07 |
| hsa05160:Hepatitis C | 17 | 2.94E-07 |
| **hsa04110:Cell cycle** | 16 | 7.68E-07 |
| hsa04668:TNF signaling pathway | 15 | 7.94E-07 |
| hsa04014:Ras signaling pathway | 21 | 9.52E-07 |
| hsa04510:Focal adhesion | 20 | 1.07E-06 |
| hsa04062:Chemokine signaling pathway | 19 | 1.16E-06 |
| **hsa04010:MAPK signaling pathway** | 22 | 1.32E-06 |
| hsa04664:Fc epsilon RI signaling pathway | 12 | 2.42E-06 |
| hsa04660:T cell receptor signaling pathway | 14 | 2.60E-06 |
| hsa04662:B cell receptor signaling pathway | 12 | 2.82E-06 |
| hsa04024:cAMP signaling pathway | 19 | 3.01E-06 |
| hsa04370:VEGF signaling pathway | 11 | 7.81E-06 |
| hsa04520:Adherens junction | 11 | 3.32E-05 |
| hsa04919:Thyroid hormone signaling pathway | 13 | 8.30E-05 |
| hsa04350:TGF-beta signaling pathway | 11 | 1.56E-04 |
| hsa04914:Progesterone-mediated oocyte maturation | 11 | 2.13E-04 |
| hsa04910:Insulin signaling pathway | 13 | 5.32E-04 |
| hsa04320:Dorso-ventral axis formation | 6 | 3.15E-03 |
| hsa04115:p53 signaling pathway | 7 | 3.98E-02 |
| | | |
| Transcription Factor Binding Sites | | |
| myogenic differentiation 1 | 149 | 6.01E-09 |
| myocyte enhancer factor 2A | 130 | 4.74E-08 |
| Interferon Regulatory Factor 7 | 125 | 1.32E-07 |
| activating transcription factor 6 | 123 | 1.74E-06 |
| forkhead box L1 | 126 | 8.84E-06 |
| forkhead box O1 | 111 | 1.03E-05 |
| MYCN proto-oncogene, bHLH transcription factor | 87 | 9.51E-05 |
| neurofibromin 1 | 98 | 2.20E-04 |
| cut like homeobox 1 | 132 | 3.41E-04 |
| myogenin / nuclear factor 1 | 115 | 1.11E-03 |

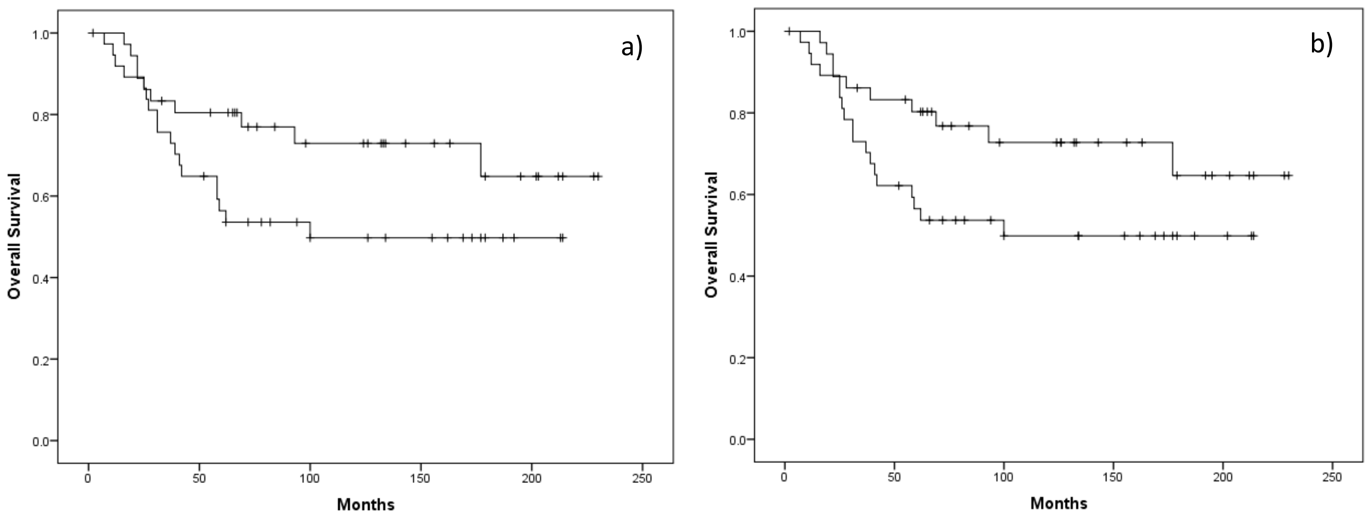


**Figure S1.** Kaplan-Meier log-rank Overall Survival analysis of candidate profiles on the MGH dataset by the supervised signed average prediction method. a) *5-miRNAs*, Median OS 100 months vs not reached, p=0.079, b) *22 miRNAs*, 100 months vs not reached, p=0.066.

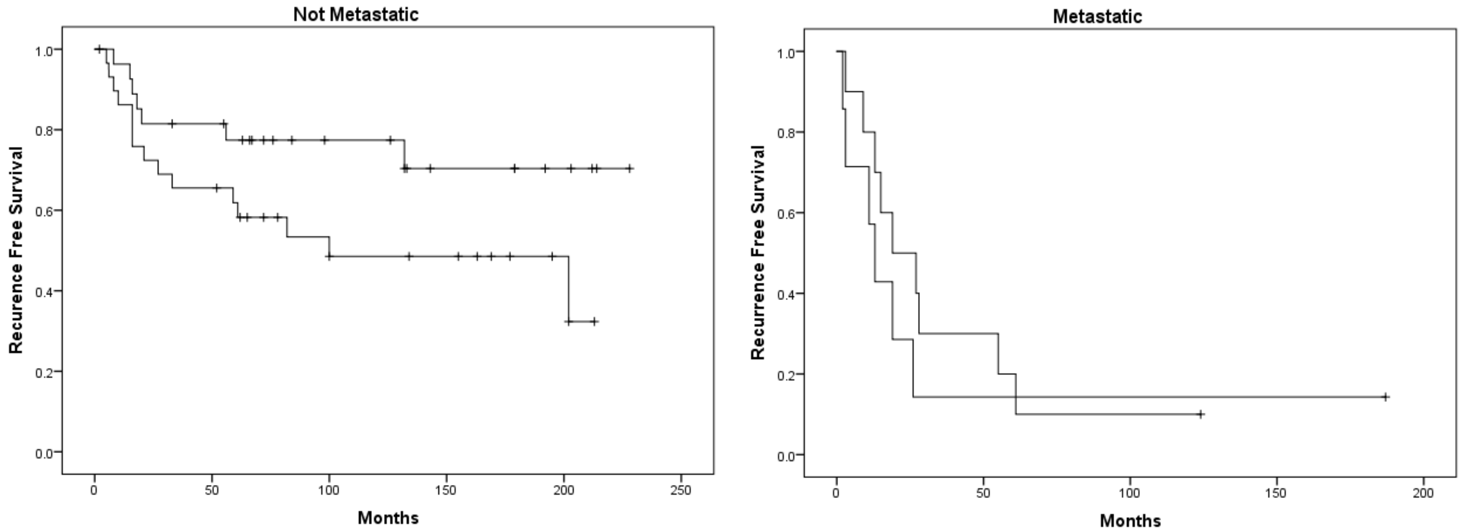


**Figure S2**. Using the 27-miRNA profile and the signed averaged supervised method, two subgroups were defined within the MGH cohort with significantly different RFS stratified for the presence of metastasis at diagnosis (median RFS 100 months vs not reached and 13 vs 19 months, stratified log rank p=0.058), supporting the previously hypothesized prognostic value of this miRNA signature (signs taken from the MGH dataset in this instance)**.**

Clustering

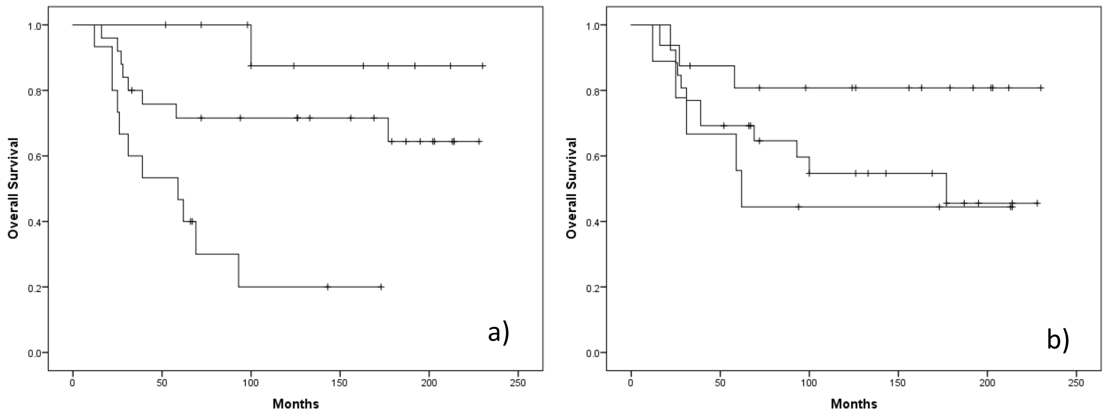


Supervised signed average prediction

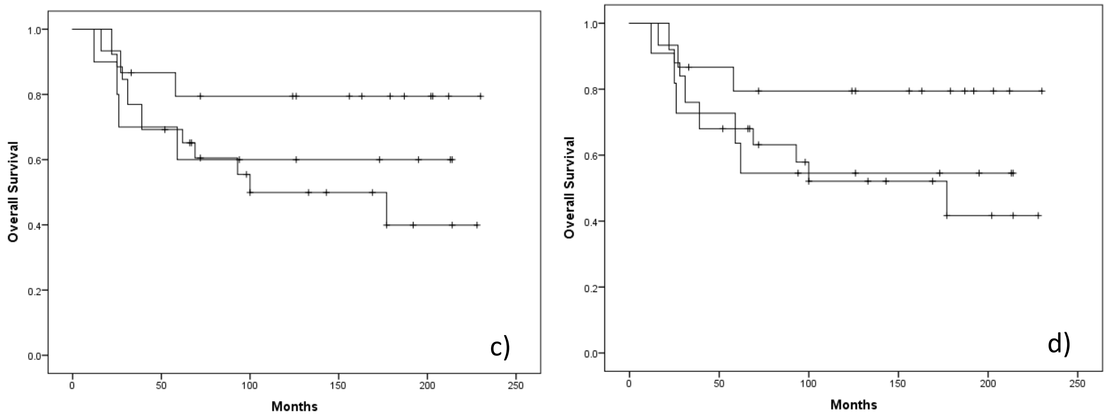


**Figure S3**. Kaplan-Meier log rank Overall Survival analysis of groups generated by a composite classification rule combining miRNA profiles and pathologically assessed chemoresponse (PCR). Three groups were defined: “Very favorable” (good prognostic profile/optimal chemoresponse, top curve), “very unfavorable” (poor prognostic profile/suboptimal chemoresponse, bottom curve) and “intermediate” (good prognostic profile/suboptimal chemoresponse, or poor prognostic profile/optimal chemoresponse, middle curve). Clustering: a) 5 miRNA-profile/PCR, Median OS 59 months vs not reached vs not reached, $p < 0.001$. b) 22 miRNA-profile/PCR, Median OS 62 months vs not reached vs not reached, $p=0.153$. Signed average: c) 5 miRNA-profile/PCR, Median OS not reached vs 100 months vs not reached, $p=0.233$. d) 22 miRNA-profile/PCR, Median OS not reached vs 177 months vs not reached, $p=0.252$.

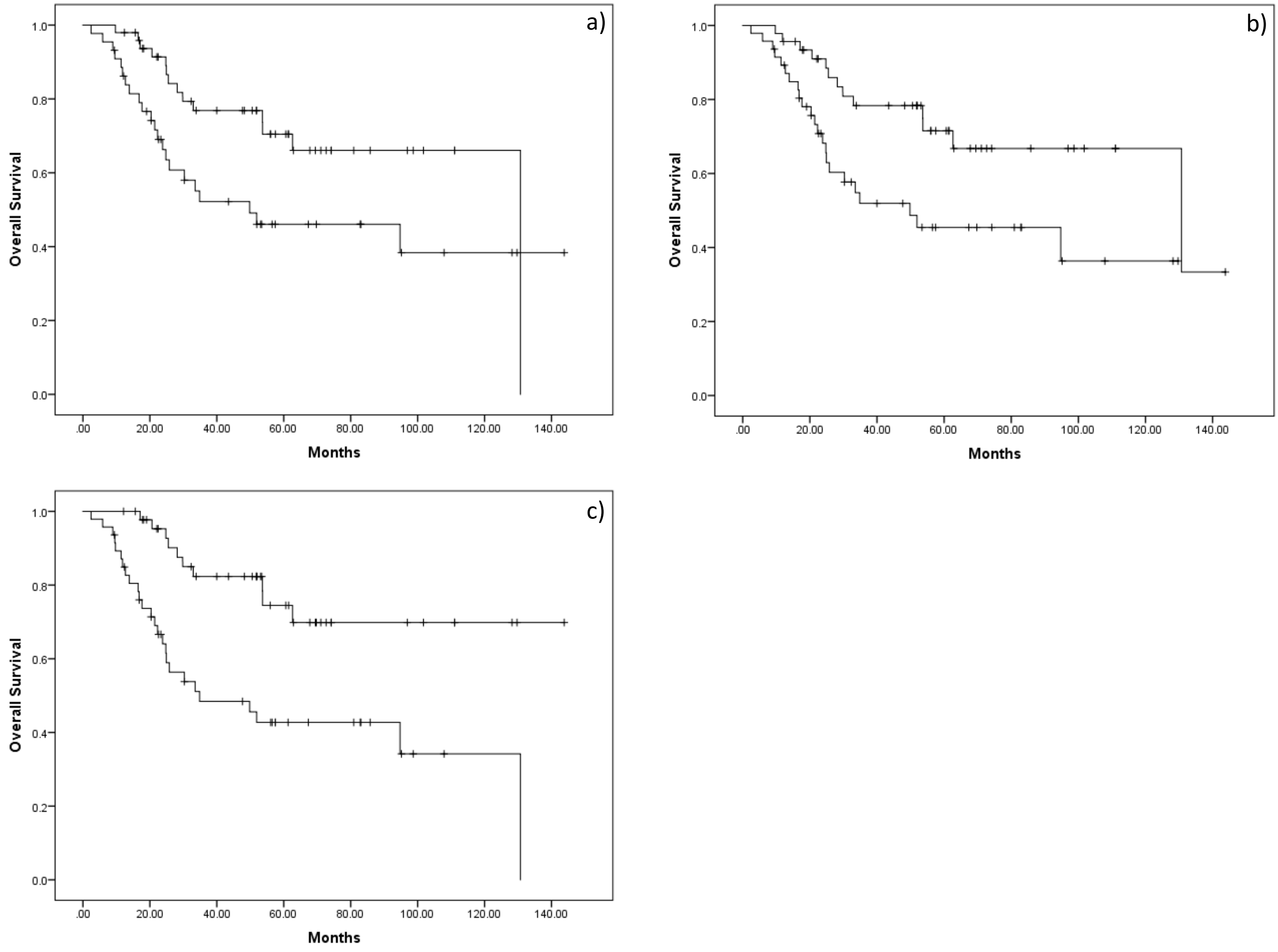


**Figure S4**. Kaplan-Meier analysis of Overall Survival groups generated by unsupervised clustering and supervised signed average analysis with the union of the restrictive list gene targets of the 5 and 22 miRNA signatures on the NCI TARGET data. a) 5-miRNA profile, unsupervised clustering, median OS 50 vs 131 months, $p = 0.017$. b) 5-miRNA profile, signed averaged, median OS 50 vs 131 months, $p = 0.008$. c) 22-miRNA profile, signed average, median OS 35 months vs not reached, $p < 0.001$.

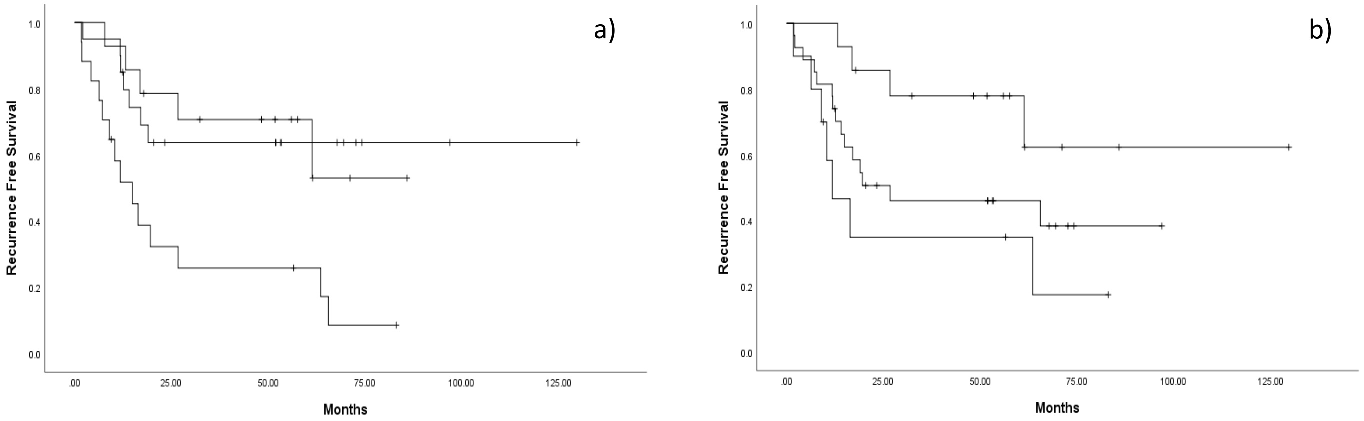


**Figure S5.** Kaplan-Meier log rank Recurrence Free Survival analysis of groups generated by a composite classification rule based on miRNA target gene profiles and pathologically assessed chemoresponse. Three groups were defined: Favorable (good prognostic profile/optimal chemoresponse, top curve), Unfavorable (poor prognostic profile/suboptimal chemoresponse, bottom curve) and Intermediate (good prognostic profile/suboptimal chemoresponse, or poor prognostic profile/optimal chemoresponse, middle curve). a) 5-miRNA profile, Median RFS 14 months vs not reached vs not reached, p=0.002. b) 22-miRNA profile, Median RFS 12 vs 27 months vs not reached, p=0.044.

**Supplementary Methods and Results**

## Supervised signed average risk prediction

A continuous supervised signed average risk score was generated with expression of the 27-miRNA profile to assess the prognostic value of this miRNA profile in the TARGET dataset.

Distribution of the risk score values: Range: -10.4673 to -5.12858, IQR: -8.9695 to -7.29871, median: -8.1818.

## Gene sets regulated by the miRNA profiles-simulations with random gene sets.

We generated 10 genesets of equal number to the union of the restrictive 5-miRNA profile target genes (n = 44) with randomly selected genes in the TARGET RNAseq dataset. We performed geneset analysis to test for association between these random genesets with survival and compared the results to those obtained with the 5-miRNA profile target genes. The list of 5-miRNA profile target genes was the only geneset significantly associated with RFS or OS (LS permutation p-value < 0.05).

| GeneSet | LS permutation p-value (RFS) | LS permutation p-value (OS) |
|---|---|---|
| *5-miRNA* target genes | 0.00311 | 0.00851 |
| Random gene list 1 | 0.12444 | 0.1291 |
| Random gene list 2 | 0.21196 | 0.22617 |
| Random gene list 3 | 0.39412 | 0.77312 |
| Random gene list 4 | 0.53484 | 0.61532 |
| Random gene list 5 | 0.59758 | 0.50063 |
| Random gene list 6 | 0.60232 | 0.55909 |
| Random gene list 7 | 0.64656 | 0.49547 |
| Random gene list 8 | 0.73606 | 0.54914 |
| Random gene list 9 | 0.76746 | 0.60586 |
| Random gene list 10 | 0.78065 | 0.97761 |

Additionally, we performed unsupervised hierarchal clustering with the 10 lists of 44 randomly selected genes in the TARGET RNAseq dataset and performed Kaplan-Meier analysis for RFS with the cluster groups. Only 1 of the 10 cluster groups generated with the random gene lists had equal or greater prognostic significance than the 5-miRNA profile target gene list by the logrank test.

| Geneset | Logrank p-value | Geneset | Logrank p-value |
|---|---|---|---|
| *5-miRNA* target genes | 0.007 | Random gene list 6 | 0.722 |
| Random gene list 1 | 0.467 | Random gene list 7 | < 0.001 |
| Random gene list 2 | 0.503 | Random gene list 8 | 0.831 |
| Random gene list 3 | 0.313 | Random gene list 9 | 0.012 |
| Random gene list 4 | 0.763 | Random gene list 10 | 0.079 |
| Random gene list 5 | 0.228 | | |
